# Attitudes toward statistics in medical postgraduates: measuring, evaluating and monitoring

**DOI:** 10.1186/1472-6920-12-117

**Published:** 2012-11-23

**Authors:** Yuhai Zhang, Lei Shang, Rui Wang, Qinbo Zhao, Chanjuan Li, Yongyong Xu, Haixia Su

**Affiliations:** 1 Department of Health Statistics and the Ministry of Education Key Lab of Hazard Assessment and Control in Special Operational Environment, School of Public Health, Fourth Military Medical University, Changle West Road 169#, Shaanxi, Xi’an, 710032, China; 2 Department of Mathematics, Fourth Military Medical University, Changle West Road 169#, Shaanxi, Xi’an, 710032, China; 3 Department of Epidemiology and the Ministry of Education Key Lab of Hazard Assessment and Control in Special Operational Environment, School of Public Health, Fourth Military Medical University, Changle West Road 169#, Shaanxi, Xi’an, 710032, China

**Keywords:** Medical postgraduate, Statistics, Survey of attitudes toward statistics

## Abstract

**Background:**

In medical training, statistics is considered a very difficult course to learn and teach. Current studies have found that students’ attitudes toward statistics can influence their learning process. Measuring, evaluating and monitoring the changes of students’ attitudes toward statistics are important. Few studies have focused on the attitudes of postgraduates, especially medical postgraduates. Our purpose was to understand current attitudes regarding statistics held by medical postgraduates and explore their effects on students’ achievement. We also wanted to explore the influencing factors and the sources of these attitudes and monitor their changes after a systematic statistics course.

**Methods:**

A total of 539 medical postgraduates enrolled in a systematic statistics course completed the pre-form of the Survey of Attitudes Toward Statistics −28 scale, and 83 postgraduates were selected randomly from among them to complete the post-form scale after the course.

**Results:**

Most medical postgraduates held positive attitudes toward statistics, but they thought statistics was a very difficult subject. The attitudes mainly came from experiences in a former statistical or mathematical class. Age, level of statistical education, research experience, specialty and mathematics basis may influence postgraduate attitudes toward statistics. There were significant positive correlations between course achievement and attitudes toward statistics. In general, student attitudes showed negative changes after completing a statistics course.

**Conclusions:**

The importance of student attitudes toward statistics must be recognized in medical postgraduate training. To make sure all students have a positive learning environment, statistics teachers should measure their students’ attitudes and monitor their change of status during a course. Some necessary assistance should be offered for those students who develop negative attitudes.

## Background

Medical statistics is an important branch of statistics. It is the application of statistical theories and methods in medical and biological research. Medical students are required to take some medical statistics courses to enable them to design, analyze and interpret experimental data in their research areas, or to make and interpret complex clinical decisions for patients. Therefore, the teaching of medical statistics is advocated during the training of all categories of medical students [1].

In China, medical statistics has traditionally been taught at the undergraduate level of medical students, usually as a course or a single unit embedded in a preventive medicine course. In the postgraduate period, medical students learn statistics in a stand-alone compulsory course that introduces systematic statistical knowledge to meet their needs and applications in both research and clinical practice. The contents of the course include descriptive statistics, some important probability distributions, some methods of estimation, hypothesis testing (t-test, analysis of variance, Chi-square test), nonparametric statistics, correlation and regression, factorial experiments, general experimental design, and the application of SPSS software. After completing a basic medical statistics course, medical postgraduates can select advanced statistics courses, such as complicated experimental design, survival analysis and multivariate statistical analysis.

Although medical statistics is very important in the training of medical students, it is considered to be a course that is difficult to teach and learn [2]. Most students have complained that statistics is more difficult than other subjects in medical training.

Many factors may influence learning statistics, such as a poor mathematical basis or background. However, statistics teachers have paid more attention to improving the cognitive aspects of instruction and little regard has been given to non-cognitive aspects, such as student attitudes toward statistics [3]. The attitudes represent a summation of emotions and feelings experienced over time in the context of learning a course. Artino [4,5] highlighted the importance of designing learning environments and confirmed that medical students’ motivational beliefs and achievement emotions were important contributors to their academic achievement. The existing literature has shown that attitudes play a crucial role in learning statistics. Students’ attitudes can hinder or assist learning statistics, especially negative ones, and can influence directly their understanding of statistical concepts and methods [6,7]. They also can affect whether students will develop useful statistical thinking skills and apply statistics knowledge in their future professional careers.

Onwuegbuzie [8] reported that between 75% and 80% of graduate students tended to develop uncomfortable levels of statistics anxiety. Green [9,10] reported a small to moderate relationship between attitudes and achievement in statistics at the post-secondary level. In our teaching experiences, negative affective responses to statistics are common among medical students who have enrolled in a statistics course. We always hear the following feelings or ideas involving this subject: “Statistics is a very difficult course”, “I was terrified when I learned statistics”, “I can’t learn this subject because my mathematics basis is very poor”, and “I will never use it, so I don’t really need to learn it”.

Individual demographic and academic factors and learning backgrounds may influence students’ attitudes toward statistics. Baloglu [11] reported that age was a factor influencing statistics anxiety and that previous mathematics experience significantly affected undergraduate students’ levels of statistics anxiety. Onwuegbuzie [12] found that gender might influence students’ attitudes toward statistics. Camona [13] and Camichael [14] also confirmed that students’ previous mathematics experience was an important source of attitudes toward statistics.

A few studies have been conducted to measure and monitor students’ attitudes in the area of statistics [3]. Several scales measuring attitudes toward statistics have been developed, such as the Attitudes Toward Statistics scale (ATS) [15] and the Survey of Attitudes Toward Statistics (SATS) [16]. The most commonly mentioned and used scale in recent studies was the SATS which was developed and validated by Schau (Additional file 1). The SATS is a cross-cultural tool and it has been confirmed and used with success internationally [13,17,18].

The existing studies have identified and measured attitudes toward statistics in high school and undergraduate students, but no one has focused on postgraduate attitudes, especially among medical postgraduates. Postgraduates have different characteristics to high school students and undergraduates, such as age, educational and professional backgrounds and research experience. To understand current attitudes held by medical postgraduates regarding statistics, we conducted a cross-sectional survey to measure these attitudes and explored their influencing factors. We also explored the relationships between the attitudes with course achievement and monitored their changes through the semester. We hope that this study may contribute to reducing student anxiety about learning medical statistics and to increasing teacher awareness of this topic.

## Methods

### Participants

The participants of this study were medical postgraduates enrolled in a medical statistics course at the Fourth Military Medical University (a large metropolitan research university). They graduated from more than 30 medical universities or colleges.

### Instruments

We adopted the SATS-28 scale to measure students’ attitudes toward medical statistics (Additional file 1). It consists of 28 seven-point Likert-type items and is divided into four subscales: Affect (feelings concerning statistics), Cognitive Competence (attitudes toward intellect and skills applied to statistics), Value (attitudes toward the usefulness and relevance of statistics) and Difficulty (attitudes toward the difficulty of the subject matter). The four subscales are formed by 6, 6, 9 and 7 items, respectively. Each item is assessed by a Likert scale with 1 =“strongly disagree” and 7 =“strongly agree” (4 is neutral). According to the directions of the instrument, the answers of some negatively worded items should be reversed (1 is replaced by 7, 2 by 6, etc.). The score of each subscale is defined as the mean score of the items that constitute the subscale. Higher scores always mean more positive attitudes. The SATS showed a good internal consistency across samples. Schau [16] and other researchers reported the Cronbach’s alpha values ranged from 0.80 to 0.89 for Affect, from 0.77 to 0.90 for Cognitive Competence, from 0.74 to 0.91 for Value, and from 0.64 to 0.86 for Difficulty [18]. Good convergent validity has been reported by relating the SATS scale scores and the Attitude Toward Statistics (ATS) scale [16,19].

The SATS-28 has two forms, a pre-form that is used before or in the beginning of a statistics course, and a post-form that is used after a statistics course. The items on both forms are identical except for some wording changes related to the timing of assessment (example items: ‘I will like statistics’ vs. ‘I like statistics’). The availability of the two forms enables comparison of attitude status at different time points in the learning process. To obtain more information about the source of the general attitude, we added an open-ended question “What are the main sources that form your general attitudes toward statistics?” The survey also asks questions about students’ demographic, educational and academic background. All information was obtained by self-report.

### Localization of the SATS-28

The original language of the two forms of the SATS-28 scale is English. In our study, they were translated into Chinese versions that were semantically and conceptually as close as possible to the original versions. A panel consisting of the authors and two translators proficient in English completed this translation task and the consensus versions were achieved. Following this, the consensus versions were tested regarding their levels of understandability, acceptability and clarity. Ten medical postgraduates, 6 men and 4 women, aged 23 to 37 years, read the Chinese versions of the SATS-28 and stated their comments. The goal of this process was to examine the face validity and cultural acceptability of the scales. In general, they stated that the Chinese versions were clear, understandable and acceptable. Nine of 10 participants were able to accurately describe what the items meant on the scales. Eight of 10 participants felt that nothing needed to be changed to make the scales easier to understand. All participants felt that there was nothing confusing and thought that it was easy to choose a response on the scales. The interviewing process showed that the scales had good face validity. Finally, according to the feedback from these participants, the panel discussed and refined the Chinese versions of the SATS-28.

### Procedure of measurement

A total of 539 medical postgraduates completed the pre-form SATS-28 scale at the second week of the medical statistics course taught in the postgraduate program (fall semester of 2009). In consideration of the logistical difficulty of the study (in completing the medical statistics course, postgraduates were separated into different departments and were difficult to convene together), 85 postgraduates (about 15%) were selected randomly from the aforementioned 539 and completed the post-form SATS-28 after the course (spring semester of 2010), and ultimately 83 of them participated in the post-course assessment. The response rate was 97.6%.

### Ethical approval

No ethical approval was required for this study according to Chinese law. However, the Ethics Committee of the Fourth Military Medical University, which reviewed and approved the project proposal, carefully considered the ethical issues concerned. There was no potential harm to participants. We obtained informed consent from all participants before conducting our study. Participants were assured that anything they selected would not influence their current or future learning. No names were registered in the survey and a control number was assigned to be used to track responses over the semester. Only one research assistant had the information that could link an individual student with their number.

### Statistical analysis

All data were analyzed using SPSS version 16 (SPSS Inc., Chicago, IL). Descriptive statistics were used to present characteristics of the study samples. One-way ANOVA and *t* test were used to compare the differences of SATS scores among demographic, educational and academic factors. Multivariable linear regression analysis was used to determine influencing factors to the attitude toward statistics. Pearson correlation coefficients were calculated to explore the relationships among the scores of the SATS and its four subscales and achievement on the examination. The paired-*t* test was used to find changes of attitudes between pre and post course. Responses to the open-ended question were analyzed to yield qualitatively distinct categories and computed percentages of frequent response types. All tests were two-tailed. *P* values ≤ 0.05 were considered significant.

## Results

### Sample characteristics

Table 1 summarizes the characteristics as well as the SATS scores of the participants. Mean age of participants was 26.5 years (M=26.5; SD=3.7, range 21–41), 57.1% were male, 73.8% had experienced 5 years of medical college training and 42.1% had taken formal systematic statistical education (as a course). Most participants (78.9%) were focused on clinical careers, including academic and nonacademic. A majority of them had not had research experience (65.5%). Most participants reported a good or neutral mathematics and computer basis or background. This meant that most participants had mastered the basic mathematics knowledge that may be used in a statistics course and had learned basic computer operating ability for some software, such as MS Excel.

**Table 1:** Main characteristics and SATS scores of participants and P values of univariable analysis (n=539)

**Characteristic**	**Number of patients (%)**	**SATS score**		*P *value*
		**M**	**SD**	
Age (years)				
<26.5	344(63.8)	4.53	0.68	<0.001
≥26.5	195(36.2)	4.20	0.62	
Sex				
Male	308(57.1)	4.42	0.69	0.79
Female	231(42.9)	4.41	0.66	
Year of medical training				
Five years	398(73.8)	4.46	0.64	<0.001
Four years	90(16.7)	4.45	0.65	
Three years	51(9.5)	4.01	0.77	
Level of statistical education				
None	50(9.3)	3.97	0.63	<0.001
A little	262(48.6)	4.31	0.67	
Systematic§	227(42.1)	4.63	0.63	
Specialty				
Clinical, nonacademic	277(51.4)	4.45	0.68	<0.001
Clinical, academic	148(27.5)	4.20	0.65	
Research, academic	84(15.6)	4.56	0.61	
Nonmedical or other‡	30(5.6)	4.71	0.70	
Research experience				
None	353(65.5)	4.32	0.66	<0.001
A little	179(33.2)	4.58	0.67	
Experienced	7(1.3)	4.82	0.62	
Mathematics basis				
Very poor	18(3.3)	3.46	0.45	<0.001
Poor	92(17.1)	4.02	0.62	
Neutral	293(54.4)	4.40	0.59	
Good	128(23.7)	4.81	0.63	
Very good	8(1.5)	5.21	0.39	
Computer basis				
Very poor	5(0.9)	4.54	0.68	<0.001
Poor	83(15.4)	4.18	0.69	
Neutral	344(63.8)	4.37	0.65	
Good	101(18.7)	4.71	0.68	
Very good	6(1.1)	4.77	0.56	

* *P* value of one-way ANOVA or *t* test.

§ Statistics has been taught as a course at the undergraduate level.

‡ Medicine or biology-related specialty, such as health management, medical electronic engineering, etc.

### Scores on SATS and subscales

Table 2 represents the mean score and standard deviations for the subscales of the SATS as well as for the entire SATS. Most medical postgraduates held positive attitudes toward statistics so that the mean score of the SATS was 4.41 (SD=0.68). Because the mean score was above neutral (mean score of 4 on a 7-point scale), we thought that the class as a group did not have an attitude problem. But the scores on the four subscales were diverse. High scores on the Affect and Cognitive Competence subscales indicated that students had positive feelings concerning statistics and had basic knowledge and skills when they learned and applied statistics. The remarkably high score on the Value subscale (M=5.45 SD=0.84) indicated that students thought statistics was very useful in their personal and professional life. Unfortunately, the Difficulty subscale had a very low score (M=2.92, SD=0.77), that is, students thought statistics was a very difficult subject.

**Table 2:** Mean scores and standard deviations for the subscales of as well as the SATS (n=539)

**Subscales**	**M**	**SD**
Affect	4.50	1.04
Cognitive Competence	4.79	0.91
Value	5.45	0.84
Difficulty	2.92	0.77
Total†	4.41	0.68

† SATS score, average of four subscales.

### The internal consistency of scores

The Cronbach’s alpha of the entire scale was estimated to be 0.86, and the values of the four subscales were estimated to be 0.82 for Affect, 0.83 for Cognitive Competence, 0.77 for Value and 0.74 for Difficulty. The values of our instrument were similar to Schau’s original scale and other studies [16,18]. Because the values range between 0.74 and 0.86, these alpha coefficients are sufficiently high to indicate scale reliability.

### Factors associated with students’ attitude toward statistics

We regressed the SATS score on pre-specified demographic, educational and academic background variables. Because there were too many variables, we conducted univariable analysis first and then included significant univariable factors into the multivariable model. The results of univariable analysis are presented in Table 1. The results showed that students’ age (age was dichotomized with mean as a cut-off), year of medical training, level of statistical education, research experience, specialty, mathematical basis, and computer basis were significantly associated with their attitudes toward statistics.

Table 3 shows the multivariable regression results for the aforementioned seven factors. Age entered the model as a binary variable, specialty entered the model as 3 dummy variables (reference category was ‘clinical, academic’), and other variables entered the model as continuous variables. The results showed that younger students had more positive attitudes toward statistics than older students. Students with higher levels of statistical education and with research experience tended to have more positive attitudes. Students with a better mathematics basis were also more positive than those with a poor basis. Moreover, with reference to ‘clinical, academic’, other categories of specialty appeared to have a positive effect on attitudes toward statistics. In other words, students with clinical academic specialties tended to have more negative attitudes than other students. Multivariate analysis confirmed most of the aforementioned univariable analysis results, but the year of medical training and computer basis became statistically nonsignificant in this model.

**Table 3:** Regression model of variables associated with students’ attitude toward statistics

Model^*^	**B**	**Beta**	**P value**
Constant	2.460		<0.001
Age	−0.154	−0.113	0.003
Year of medical training	0.061	0.039	0.122
Level of statistical education	0.191	0.185	<0.001
Research experience	0.098	0.075	0.041
Mathematics basis	0.329	0.387	<0.001
Computer basis	0.074	0.073	0.057
Specialty†			
Clinical, nonacademic	0.159	0.120	0.007
Research, academic	0.198	0.109	0.014
Nonmedical or other	0.291	0.101	0.012

R square of the model is 0.41.

*Age entered the model as a binary variable, Specialty entered the model as 3 dummy variables, and other variables entered the model as continuous variables.

†Reference category was ‘clinical, academic’.

### The relationship between students’ attitudes and course achievement

At the end of the semester, students took a final examination of the medical statistics course. The examination was objective and based on quantitative criteria. Thus, achievement on the examination could be used as an indicator to evaluate the level of knowledge mastery. The mean mark on the examination was 72.7 (SD=11.1, hundred mark system).

Table 4 presents the Pearson correlation coefficients among the scores of the SATS and its four subscales and achievement on the examination. There was a positive correlation between achievement on the examination and SATS score (r=0.44, 95% CI: 0.37-0.51), that is, students with more positive attitudes toward statistics tended to perform better on the examination. Moreover, the achievement was moderately related to the Affect (r=0.40, 95% CI: 0.33-0.47), Cognitive Competence (r=0.43, 95% CI: 0.36-0.50) and Value (r=0.32, 95% CI: 0.24-0.39) subscales. Although the correlation between achievement and the Difficulty subscale was also significant (r=0.17, 95% CI: 0.09-0.25), the small correlation coefficient indicated that there was no substantial correlation between them.

**Table 4:** Correlations among the SATS, subscales scores and the achievement of examination

	**Affect**	**Cognitive Competence**	**Value**	**Difficulty**	**SATS**
Cognitive Competence	0.74^*^				
Value	0.49^*^	0.49^*^			
Difficulty	0.37^*^	0.40^*^	0.03		
SATS	0.86^*^	0.86^*^	0.73^*^	0.55^*^	
Achievement	0.40^*^	0.43^*^	0.32^*^	0.17^*^	0.44^*^

*Pearson correlation coefficients were significant at p<0.05.

The inter-relationships among subscales were all positive. The Affect and the Cognitive Competence subscales were strongly related to each other. The Value and the Difficulty subscales were moderately related to the Affect and Cognitive Competence subscales, but were not related to each other. Students seemed to value statistics regardless of the perceived difficulty.

### The sources of students’ attitudes toward statistics

According to the open-ended question about the source of the general attitude toward statistics, we concluded that the students’ attitudes mainly came from experiences in the former statistical or mathematical class (63.1%, 340/539). Some students believed statistics was a part of mathematics and so their attitudes toward mathematics were merely transferred to statistics. Other sources included colleagues’ and senior students’ influence (18.9%, 102/539) and students’ previous experiences of the application of statistics in medical research or reading literature (7.6%, 41/539). In 10.4% (56/539), the students answered other varied sources, such as out-of-school lives.

### Continuous monitoring of students’ attitudes toward statistics

The results of 83 paired data showed that there were only slight changes on the attitudes after completing the medical statistics course (Table 5). All the changes were negative. The SATS score decreased 0.28 points (M=−0.28, SD=0.32, about 6.4%). The Affect, Cognitive Competence and Difficulty scores showed significant negative changes, while the change on the Value score was non-significant. The Affect score showed the largest change (M=−0.30, SD=0.80), which indicated students developed more stressful and uncomfortable feelings after the course. The negative change of Cognitive Competence (M=−0.22, SD=0.81) and Difficulty scores (M=−0.26, SD=0.83) indicated that students encountered more troubles and perceived more difficulties in statistics. The non-significant very minor change of the Value score (M=−0.09, SD=0.70) indicated that students still felt statistics was very useful in their professional career and everyday life.

**Table 5:** Changes of attitudes between pre and post course (n=83)

**Scales**	**Mean Difference**	**SD (paired)**	**t-value (paired)**	**P value**
Affect	−0.30	0.80	−3.416	0.001
Cognitive Competence	−0.22	0.81	−2.511	0.014
Value	−0.09	0.70	−1.190	0.238
Difficulty	−0.26	0.83	−2.903	0.005
SATS	−0.28	0.32	−7.974	<0.001

## Discussion

The results of this study suggest that medical postgraduates hold relatively positive attitudes toward statistics with the exception of the difficulty aspect. Unlike other studies, the participants in our study were postgraduates who knew more about their professional requirements. Many participants had taken an introductory statistics course and had experience of the application of statistics. These characteristics may help them to form positive attitudes toward statistics, especially in terms of the value aspect.

We confirmed that some demographic and academic factors and learning backgrounds could affect students’ attitudes toward statistics. We found that younger postgraduates held more positive attitudes than older ones. The result was consistent with other studies that demonstrated attitude differences between age groups, although those studies were based on different students [11,20]. The older students may encounter more difficulties than younger students in understanding and grasping statistics. We did not find attitude differences in gender, whereas Onwuegbuzie [12] found that women’s attitudes were more negative than men’s. The results about gender in the published literature are not consistent. About one half of the studies found attitude differences by gender, while the other one half did not [21]. We found that research experience affected students’ attitudes toward statistics. It may be that students with research experience have realized the value of statistics and have grasped some ability with basic statistical analysis during their research. The influences of students’ statistical education level and mathematics basis on their attitudes have been confirmed by similar studies [3,13]. Students who experienced a higher level of statistical education or had a better mathematics basis tended to hold a more positive attitude toward statistics. In the literature about statistics education, it is generally agreed that one important source of the attitudes toward statistics is previous experience in mathematics [3,13,14]. Unexpectedly, we found that specialty was a significant influencing factor associated with students’ attitudes toward statistics. The attitudes of students with clinical academic specialties were the worst. It was an interesting phenomenon and the mechanism of influence was ambiguous. Although computer basis was no longer significant in the multivariable model, there was a positive correlation trend between computer basis and attitude. Statistics concerns complicated computations, therefore, students with a better computer basis should have more confidence in the computational aspects of statistics. The year of medical training was also nonsignificant in the multivariable model, but the students with only three years of medical training held more negative attitudes than those with longer medical training.

We found that achievement on the examination was positively related to the attitude toward statistics with the exception of the difficulty aspect. It confirmed that positive and effective attitudes can be associated with improved student statistics performance. These findings were consistent with those obtained by Schau, who reported similar relationships between course grade and attitude scores on the Affect, Cognitive Competence, and Value subscales of the SATS [16]. However, other studies found that the Value subscale was not related with course achievement [13,22]. An interesting finding was the very weak relationship between the Difficulty subscale and achievement on the examination. It is generally believed that students who perceive more difficulties in learning seem to obtain worse course achievement, but our study did not confirm this supposition. Might students who perceive more difficulties make more efforts to study?

We found that the global attitude and its components presented negative changes after the course except for the value aspect, although some students already had experience of learning statistics at the graduate level. This may be attributed to teaching quality to a certain extent, such as improper teaching methods, but we thought that this was not the main reason. In our opinion, these negative changes may be as the result of students learning more advanced statistical knowledge in the postgraduate statistics course. This more complex statistical knowledge influenced the students’ feelings and confidence with statistics. Although they encountered more difficulties, postgraduates still recognized the value of statistics in their future professional careers. This perhaps explains the non-significant very minor change in the value aspect of statistics. Schau found similar negative changes in his study although the study was not paired [23]. Thus it can be seen that these negative changes were not special cases.

Improving students’ attitudes toward statistics may have a direct positive effect on learning statistics. Accordingly, an important issue for teachers is how to improve students’ attitudes toward statistics, especially, how to reduce the fear and anxiety in learning statistics. Many studies have explored the feasibility of improving students’ attitudes by establishing innovative pedagogical strategies, such as using course-related materials, journal writing, and avoiding the use of statistical terminology and complex statistical formulae [8,17,22,24]. Several studies have also demonstrated that the use of advanced technology can enhance learning environments, such as adopting integrated technologies and using an online environment [25-27]. In our teaching experiences, we thought that both innovative pedagogical strategies and advanced technologies may improve students’ attitudes toward statistics to a certain extent, but substantially reducing students’ stress, anxiety and frustration with statistics is difficult.

## Conclusions

Statistical knowledge and skills are essential for medical postgraduates’ academic or professional careers. Our findings emphasize the importance of students’ attitudes toward statistics in the medical postgraduate training process because they can influence the learning of statistics significantly. The characteristics of medical postgraduates resulted in different responses to statistics from other types of students. Teachers should pay more attention to these characteristics and provide appropriate curriculum adjustments or interventions for students with negative attitudes. In particular, more effective interventions are needed to help students overcome their fear and anxiety toward statistics. Future studies should consider developing and testing such interventions.

### Limitations

This study surveyed medical postgraduates at a single institution and had a small sample size. These limitations may affect the generalizability of our findings although the participants in our study represented a broad range of medical education backgrounds from around China.

## Abbreviations

SATS: Survey of Attitudes Toward Statistics; ATS: Attitudes Toward Statistics scale; SPSS: Statistical Package for the Social Sciences; ANOVA: Analysis of variance.

## Competing interests

The authors declare that they have no competing interests.

## Authors’ contributions

YZ, LS, YX and HS designed and conducted the study, RW, QZ and CL collected the data and conducted the statistical analysis. All four authors contributed to the writing of the paper and approved of the final manuscript. All authors read and approved the final manuscript.

## Pre-publication history

The pre-publication history for this paper can be accessed here:

http://www.biomedcentral.com/1472-6920/12/117/prepub

## Supplementary Material

Additional file 1 **The SATS-28 is available from the author: Candace Schau (CS Consultants, LLC, Albuquerque, NM 87111; cschau@comcast.net).** The pretest versions of the SATS-28 can be viewed at: http://www.evaluationandstatistics.com. Click here for file

## References

[B1] Hunponu-Wusu OO The need for medical statistics in the training of health personnel Med Educ 1977 11 351 354 10.1111/j.1365-2923.1977.tb00627.x 904498

[B2] Garfield JB Assessing statistical reasoning Stat Educ Res J 2003 2 22 38

[B3] Gal I Ginsburg L Schau C Gal I, Garfield JB Monitoring attitudes and beliefs in statistics education The assessment challenge in statistics education 1997 INetherlands: OS Press 37 51 http://www.stat.auckland.ac.nz/~iase/publications/assessbkref

[B4] Artino AR Holmboe ES Durning SJ Can achievement emotions be used to better understand motivation, learning, and performance in medical education? Med Teach 2012 34 240 244 10.3109/0142159X.2012.643265 22364457

[B5] Artino AR La Rochelle JS Durning SJ Second-year medical students’ motivational beliefs, emotions, and achievement Med Educ 2010 44 1203 1212 10.1111/j.1365-2923.2010.03712.x 21091760

[B6] Onwuegbuzie AJ Modeling statistics achievement among graduate students Educ Psychol Meas 2003 63 1020 1038 10.1177/0013164402250989

[B7] Zeidner M Statistics and mathematics anxiety in social science students: some interesting parallels Br J Educ Psychol 1991 61 319 328 10.1111/j.2044-8279.1991.tb00989.x 1786211

[B8] Onwuegbuzie AJ Attitudes toward statistics assessments Assessment Eval in High Educ 2000 25 321 339 10.1080/713611437

[B9] Green KE The affective component of attitude in statistics instruction 1994, April New Orleans: In current trends and issues in teaching statistics. Symposium conducted at the meeting of the American Educational Research Association

[B10] Waters LK Martelli TA Zakrajsek T Popovich P Attitudes toward statistics: an evaluation of multiple measures Educ Psychol Meas 1988 48 513 516 10.1177/0013164488482026

[B11] Baloglu M Individual differences in statistics anxiety among college students Pers Indiv Differ 2003 34 855 865 10.1016/S0191-8869(02)00076-4

[B12] Onwuegbuzie AJ Statistics test anxiety and female students Psychol Women Quart 1995 19 413 418 10.1111/j.1471-6402.1995.tb00083.x

[B13] Carmona J Martínez RJ Sánchez M Mathematical background and attitudes toward statistics in a sample of Spanish college students Psychol Rep 2005 97 53 62 16279305 10.2466/pr0.97.1.53-62

[B14] Carmichael C Callingham R Watson J Hay I Factors influencing the development of middle school students’ interest in statistical literacy Stat Educ Res J 2009 8 62 81

[B15] Wise SL The development and validation of a scale measuring attitudes toward statistics Educ Psychol Meas 1985 45 401 405

[B16] Schau C Stevens J Dauphinee TL Del Vecchio A The development and validation of the Survey of Attitudes Toward Statistics Educ Psychol Meas 1995 55 868 875 10.1177/0013164495055005022

[B17] Cherney ID Cooney RR Predicting student performance in a statistics course using the mathematics and statistics perception scale (MPSP) T Nebraska Acad Sci 2005 30 1 8

[B18] Chiesi F Primi C Assessing statistics attitudes among college students: Psychometric properties of the Italian version of the Survey of Attitudes toward Statistics (SATS) Learn Individ Differ 2009 19 309 313 10.1016/j.lindif.2008.10.008

[B19] Cashin SE Elmore PB The Survey of Attitudes Toward Statistics Scale: a construct validity study Educ Psychol Meas 2005 65 509 524 10.1177/0013164404272488

[B20] Köller O Baumert J Schnabel K Does interest matter? The relationship between academic interest and achievement in mathematics J Res Math Educ 2001 32 448 470 10.2307/749801

[B21] Hilton SC Schau C Olsen JA Survey of attitudes toward statistics: factor structure invariance by gender and by administration time Struct Equ Model 2004 11 92 109 10.1207/S15328007SEM1101_7

[B22] Schutz PA Drogosz LM White VE Distefano C Prior knowledge, attitude and strategy use in an introduction to statistics course Learn Individ Differ 1998 10 291 308 10.1016/S1041-6080(99)80124-1

[B23] Schau C Students’ attitudes: The “other” important outcome in statistics education ASA Proceedings: Papers presented at the American Statistical Association Joints Statistical Meetings 2003 Alexandria, VA: American Statistical Association, Section on Statistical Education. [CD-ROM] 3673 3681

[B24] Smith CH Miller DM Robertson AM Using writing assignments in teaching statistics: an empirical study Math Comput Educ 1992 26 21 34

[B25] Meletiou-Mavrotheris M Lee C Fouladi RT Introductory statistics, college student attitudes and knowledge - A qualitative analysis of the impact of technology-based instruction Int J Math Educ Sci Technol 2007 38 65 83 10.1080/00207390601002765

[B26] Schou SB A study of student attitudes and performance in an online introductory business statistics class Electron J Integr Technol Educ 2007 6 71 78

[B27] Suanpang P Petocz P Kalceff W Student attitudes to learning business statistics: comparison of online and traditional methods Educ Technol Soc 2004 7 9 20

