# Supplementary material for: Attitudes toward statistics in medical postgraduates: measuring, evaluating and monitoring
Source: BMC Med Educ. 2012 Nov 23;12:117. doi: 10.1186/1472-6920-12-117 (PMC3533942; doi:10.1186/1472-6920-12-117)
Supplement: Additional file 1 — The SATS-28 is available from the author: Candace Schau (CS Consultants, LLC, Albuquerque, NM 87111; cschau@comcast.net). The pretest versions of the SATS-28 can be viewed at: http://www.evaluationandstatistics.com. [file 1472-6920-12-117-S1.pdf]

理文编辑
edanz group china

15/10/2012

To whom it may concern,

Re: "Attitudes toward statistics in medical postgraduates: measuring, evaluating and monitoring"

By Haixia Su (Department of Epidemiology and the Ministry of Education Key Lab of Hazard Assessment and Control in Special Operational Environment, School of Public Health, Fourth Military Medical University, Changle West Road 169#, Xi'an, Shaanxi, 710032, China.)

This is to confirm that this text has been edited by Thomas Laage (MD), an editor working for Liwen Bianji (Edanz Group China).

Sincerely yours,

Benjamin Shaw
Global and China Director
Liwen Bianji (Edanz Group China)

EDANZ EDITING CHINA
北京精锐理文文化传播有限公司

Liwen Bianji (Edanz Group China)
Interchina Commercial Building, 1112A
No 33. Dengshikou Street, Dongcheng District, Beijing, P.C. 100006, China
TEL +86-10-6528-0877 FAX +86-10-6528-0834 EMAIL editing@liwenbianji.cn
